# Chlorhexidine and benzalkonium chloride: promising adjuncts in combating multidrug resistant *Klebsiella pneumoniae* in healthcare settings

**DOI:** 10.1186/s12879-025-10980-w

**Published:** 2025-05-07

**Authors:** Amal F. Makled, Azza Z. Labeeb, Heba M. Moaz, Asmaa S. Sleem

**Affiliations:** Department of Medical Microbiology & Immunology, Faculty of Medicine, Menoufia University, Shibīn al Kom, Egypt

**Keywords:** *Klebsiella pneumoniae*, Chlorhexidine, Benzalkonium chloride, *qacE∆1*, *CepA*

## Abstract

**Background:**

Hospital-acquired infections caused by multidrug resistant (MDR) *Klebsiella pneumoniae* pose a significant global health threat. Effective antisepsis and disinfection protocols are mandatory to prevent these infections. This study aimed to isolate *Klebsiella pneumoniae*, evaluate antimicrobial susceptibility, and assess the efficacy of selected biocides.

**Methods:**

Fifty clinical MDR *Klebsiella pneumoniae* isolates were collected from various hospital departments. Antimicrobial susceptibility was determined using the disc diffusion method. Minimum inhibitory concentrations (MICs) of chlorhexidine and benzalkonium chloride were measured via agar dilution. Conventional PCR was employed to detect biocide resistance genes (*qacE∆1* and *cepA*).

**Results:**

*Klebsiella pneumoniae* was identified in 19.16% of cases. All isolates exhibited multidrug resistance, with multiple antimicrobial resistance indices ranging from 0.24 to 0.92, reaching up to 1. Benzalkonium chloride MICs significantly increased with resistance, reaching up to 64 µg/mL, while chlorhexidine MICs were consistent across isolates. The *qacE∆1* and *cepA* genes were detected in 62% and 72% of isolates, respectively, with a significant association between *qacE∆1* and cephalosporin resistance. No significant correlation was found between biocide MICs and clinical specimen types or hospital units.

**Conclusion:**

The *cepA* gene is closely associated with extensive drug resistance in *Klebsiella pneumoniae*, emphasizing its role in antimicrobial resistance. Optimized biocide formulations, when properly developed and applied, can play a crucial role in combating and preventing infections caused by multidrug-resistant *Klebsiella pneumoniae*.

**Supplementary Information:**

The online version contains supplementary material available at 10.1186/s12879-025-10980-w.

## Clinical trial number

Not applicable.

**Supplementary Information:**

The online version contains supplementary material available at 10.1186/s12879-025-10980-w.

## Introduction

*Klebsiella pneumoniae*, listed by the World Health Organization as a priority pathogen, is infamous for driving multidrug resistant (MDR) healthcare associated infections. Controlling the spread of *K. pneumoniae* requires ongoing assessment and advancement of effective antimicrobial strategies, including both antibiotics and biocides [1]. Biocides, including skin antiseptics and surface disinfectants, play a vital role in healthcare settings by reducing microbial bioburden on hospital personnel and within the environment. As prevention is often more effective than treatment, the development and application of biocidal agents with broad-spectrum bactericidal activity remain essential priorities, particularly in combating drug resistant *K. pneumoniae* [2, 3].

Chlorhexidine (CHX), a widely used biocide, is incorporated into cosmetics and pharmaceutical products such as eye drops, wound dressings, and antiseptic washes. CHX demonstrates activity against Gram-positive and Gram-negative bacteria, facultative anaerobes, aerobes, and yeasts. Its bactericidal action arises from its cationic nature, which enables it to bind to negatively charged bacterial cell walls, causing membrane disruption. At low concentrations, CHX exhibits bacteriostatic effects, while at higher doses, it becomes bactericidal [4]. Similarly, benzalkonium chloride, a member of the quaternary ammonium compounds (QACs), is a cationic surfactant with notable bactericidal and fungicidal properties. QACs are widely used due to their pleasant odor and low toxicity. Their primary mechanism of action involves disrupting the structure and function of cell membranes, with biocidal efficacy varying significantly depending on concentration [5].

Concerns about bacterial resistance to biocides are growing, paralleling the global challenge of antibiotic resistance [6]. The excessive use of antibiotics and biocides has exerted selective pressure on bacterial strains, leading to the widespread distribution of resistance genes. Biocide resistance genes, such as *qacA/B*,* qacE*,* qacEΔ1*,* cepA*, and *arcA*, have been identified in multidrug resistant (MDR) bacteria [7].

The *cepA* gene, which encodes a chlorhexidine efflux pump, is associated with chlorhexidine resistance in Gram-negative bacteria, particularly in *Klebsiella* spp [1]. Similarly, quaternary ammonium compound (QAC) resistance genes *(qac)* produce efflux pumps that actively expel biocides from bacterial cells, reducing their intracellular antimicrobial effectiveness [8]. Alarmingly, MDR strains have demonstrated cross-resistance to biocides, likely due to shared resistance mechanisms, further complicating the fight against hospital-acquired bacterial infections [9].

**The objective of this study** was to explore the relationship between the presence of biocide resistance genes (*qacE∆1 and cepA*) and the minimal inhibitory concentrations (MICs) of chlorhexidine and quaternary ammonium compounds in MDR *Klebsiella pneumoniae* with the goal of advancing strategies to control MDR *Klebsiella* infections.

## Patients and methods

### Study design and ethical consideration

This study was performed in Medical Microbiology and Immunology Department, Faculty of medicine, Menoufia University during the period from May 2022 to June 2024.Totally two hundred- eighty four clinical samples were collected from hospitalized patients with evident infections 48 h or more after hospital admission. Patients from Intensive care units (ICUs) and different hospital departments were included. Different age groups were involved. Clinical and demographic data were collected and analyzed, considering patients’ age, gender, hospital stay duration, antibiotic uptake, comorbidities, and exposure to invasive procedures. The study protocol was approved by the local Ethics Committee of the Faculty of Medicine, Menoufia University (**IRB: 3/2022MICRO44**).

### Clinical sample collection and transport

Different clinical samples were collected aseptically based on the infection site: wound and burn swabs, respiratory samples (sputum and endotracheal aspirates), mid-stream urine from non-catheterized patients, catheter port urine from catheterized patients, and blood samples into culture bottles (bioMerieux, France). Each sample was carefully labeled and promptly transported to the microbiology laboratory for rapid processing.

### Isolation and identification of Klebsiella spp

All clinical samples were inoculated onto conventional media obtained from Oxoid, UK for the isolation and identification of *Klebsiella* spp., and CLED media for urine bacterial counting, followed by aerobic incubation at 37 °C for 24–48 h. The phenotypic identification of *Klebsiella* isolates was based on colony morphology and biochemical reactions [10]. Further confirmation and species identification were performed using the VITEK-2 Compact System- Biomerieux, France, where fifty *Klebsiella pneumoniae* isolates were verified.

### Antimicrobial susceptibility testing (AST) of Klebsiella pneumoniae isolates

Antimicrobial susceptibility testing of *Klebsiella pneumoniae* isolates was performed using the Kirby-Bauer disk diffusion method on Muller-Hinton agar, with results interpreted according to CLSI 2023 guidelines. Various antibiotic discs (Oxoid, UK) were tested as demonstrated later, including ampicillin, carbapenems, cephalosporins, aminoglycosides, tetracyclines, macrolides, fluoroquinolones, trimethoprim-sulfamethoxazole and nitrofurantoin exclusively for urine samples ***(CLSI***,*** 2023).*** On the same line, Colistin(Sigma, Egypt). MIC was specifically tested through the agar dilution method considered as critical last-resort antibiotic option against multidrug resistant *Klebsiella* [11]. The Multiple Antimicrobial Resistance (MAR) index that quantifies the resistance of bacterial isolates to various antimicrobial agents was calculated by dividing the number of antibiotics to which an isolate is resistant by the total number of antibiotics tested [12].

### Determination of the minimum inhibitory concentrations (MICs) of biocides

The MICs for benzalkonium chloride and chlorhexidine were determined using the agar dilution method. Benzalkonium chloride (LOBA chemical, India– 50 gm/100 ml solution) was serially diluted in Muller-Hinton agar to achieve a concentration range of 0.125 to 1024 µg/ml. Chlorhexidine HCL (125 mg/100 ml - Hexitol, Egypt) was similarly diluted. Prepared agar plates were incubated at 37 °C, and the MIC was determined as the lowest concentration of biocide at which no visible colony growth of studied *Klebsiella* isolate was observed [11].

### Molecular detection of biocide resistance genes

Conventional polymerase chain reaction (PCR) was employed to detect the presence of biocide resistance genes *qacE∆1* and *cepA*. DNA was extracted using the Thermo Fisher GeneJET purification kit and stored at -20 °C. PCR amplification was performed using T professional Thermocycler (Biometra, Germany) with specific primers for *qacE∆1* and *cepA* resistance genes. The optimized PCR protocol is illustrated in Table 1. The amplified products were analyzed by electrophoresis on a 1.5% agarose gel, run at 100 volts for one hour. Gene presence was confirmed by visualization under a UV trans-illuminator, with bands appearing at 190 bp for *qacE∆1*and 1051 bp for *cepA* [13, 14].

**Table 1:** Primer sequences and thermal cycling conditions of *QacE∆1* and *CepA* resistance genes

Gene	Primer sequence (5’ → 3’)	Thermal cycling conditions						Amplicon size (bp)
		Initial denaturation (°C/time)	Denaturation (°C/time)	Annealing (°C/ time)	Cycles	Extension (°C/time)	Final extension	
***qacE∆1***	F: AATCCATCCCTGTCGGTGTT R: CGCAGCGACTTCCACGATGGGGAT	94 /5 min	94/30 sec.	53/45 sec.	30	72/50 sec.	72/10 min.	190
***cepA***	F: CAACTCCTTCGCCTATCCCG R: TCAGGTCAGACCAAACGGCG	94/5 min.	94/30 sec.	66/45 sec.	30	72/50 sec.	72/10 min.	1051

### Statistical analysis

Data coding, validation and analysis were conducted by the Statistical Package for the Social Sciences (SPSS), version 20 (SPSS Inc., Chicago, IL, USA). Continuous variables are expressed as mean, median, ranges and SD. Categorical variables are expressed as frequencies and percentages. Chi square, ANOVA and Kruskal wallis tests were used. A significance level of *P* < 0.05 was used in all tests.

## Results

Among 261 growing microbial isolates, fifty *Klebsiella pneumoniae* strains (19.16%) were identified added to other pathogens such as *Pseudomonas spp.*, *Staphylococcus aureus* and *Acinetobacter spp.* (Supplementary Tables 1-S). The study population comprised 144 males (55.2%) and 117 females (44.8%), with a mean age of 40.59 ± 22.66 years. Remarkably, 86.3% of the patients had prior antibiotic exposure, and 79% experienced hospital stays lasting seven days or more. Additionally, 74% underwent invasive procedures, highlighting the intensive nature of medical interventions. Comorbidities were present in 68% of cases, reflecting significant underlying health challenges that likely contributed to extended hospitalizations.

**Fig. 1:**
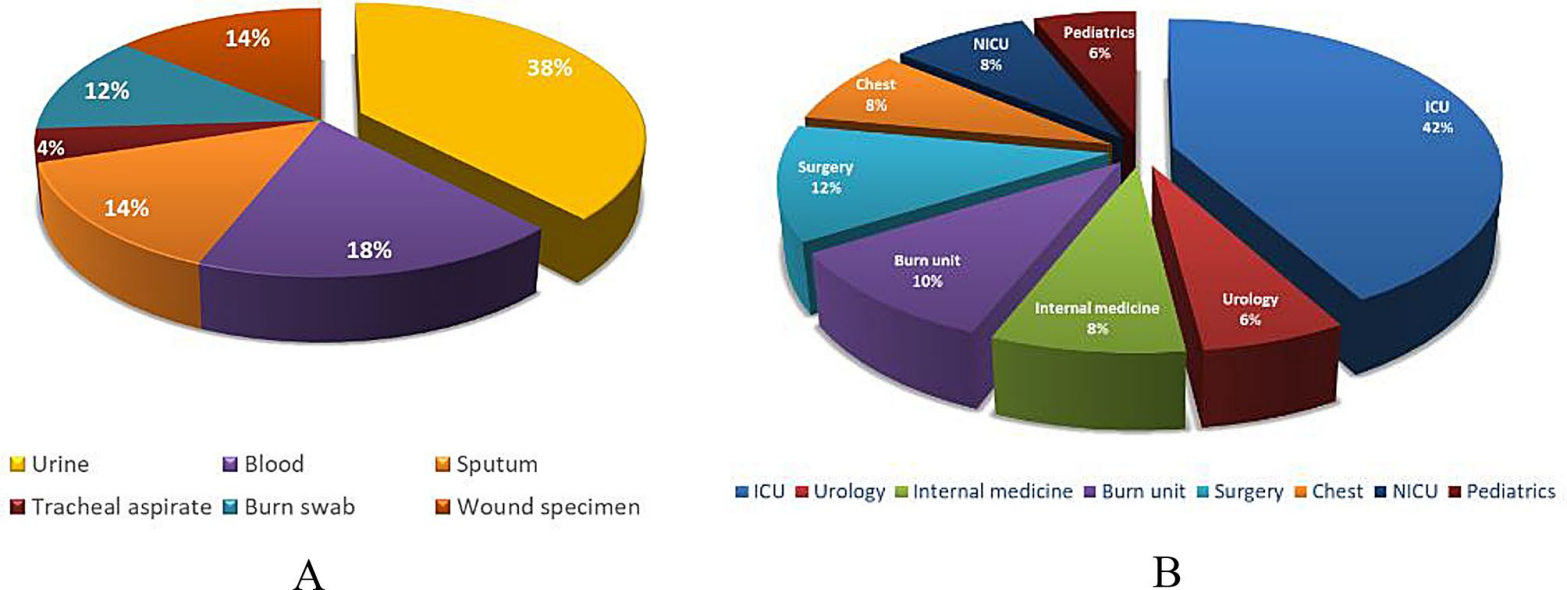
Distribution of *Klebsiella pneumoniae* Isolates Across Specimens and Departments. (**A**): Specimen-wise Distribution of *klebsiella pneumoniae* isolates. (**B**): Department-wise Distribution of *klebsiella pneumoniae isolates*

Urine samples were the most common source of *Klebsiella pneumoniae* (38%), followed by blood samples (18%), sputum specimens (14%), burn swabs and wound specimens (12% and 14%, respectively), while tracheal aspirates contributed only 4% of *Klebsiella* isolates (Fig. 1-a). Intensive care units recorded the highest number of isolates by 42%, followed by surgery department (12%) and burn unit (10%). Each of internal medicine department, chest department and NICU accounted for 8% of isolates, while both of urology department and pediatrics department contributed for 6% each (Fig. 1-b).

Resistance patterns among *Klebsiella* isolates revealed significant variability. High resistance rates were observed for ampicillin (84%) and tested cephalosporins (≥ 82%). Resistance was similarly high for macrolides (azithromycin, 90%) and quinolones (ciprofloxacin, 78%; levofloxacin, 76%). Surprisingly, newer combinations like ceftazidime-avibactam and piperacillin-tazobactam also showed resistance rates of 70% and 68%, respectively. Conversely, carbapenems demonstrated notable effectiveness, with meropenem showing 80% susceptibility, followed by imipenem (70%) and ertapenem (62%). For urinary isolates, nitrofurantoin exhibited a moderate resistance rate (47.3%). Regarding colistin, most isolates (88%) were intermediate-sensitive (MIC ≤ 2 µg/mL), with only 12% classified as resistant (MIC ≥ 4 µg/mL) following **CLSI 2023** guidelines [11] (Supplementary Tables 2-S).

Twenty four *Klebsiella* isolates (48%) exhibited multidrug resistance (MDR) with MAR indices ranging from 0.24 to 0.71. Extensively drug resistance (XDR) was observed in 40% of isolates (MAR indices: 0.75–0.92), while pan-drug resistance (PDR) with one whole MAR index was noted in 12% of *Klebsiella* isolates with observed resistance to all tested antibiotics, including colistin (Supplementary Tables 3-S & 4-S).

**Fig. 2:**
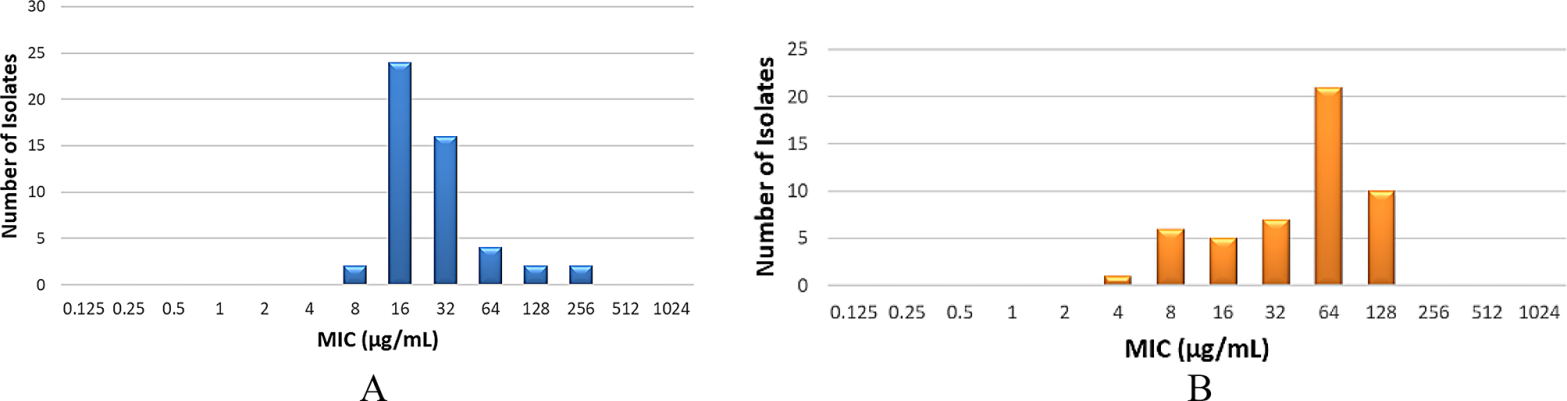
Benzalkonium Chloride & Chlorhexidine Minimal Inhibitory Concentrations (MIC) Distribution Among *Klebsiella pneumoniae* Isolates. (**A**): Benzalkonium Chloride MIC Distribution among isolates. (**B**): Chlorhexidine MIC Distribution among isolates

For biocides, benzalkonium chloride MIC values ranged from 8 to 256 µg/mL, with MIC50 at 16 µg/mL and MIC90 at 64 µg/mL. Chlorhexidine MIC values ranged from 4 to 128 µg/mL, with MIC50 and MIC90 at 64 µg/mL and 128 µg/mL, respectively (Fig. 2-a & 2-b). MDR and XDR strains displayed consistent median MIC values (16 µg/mL) for benzalkonium chloride, whereas PDR strains required significantly higher concentrations (64 µg/mL). This difference was statistically significant (*p* = 0.005). In contrast, chlorhexidine MIC values did not vary significantly across different resistance phenotypes (*p* = 0.670) (Table 2).

**Fig. 3:**
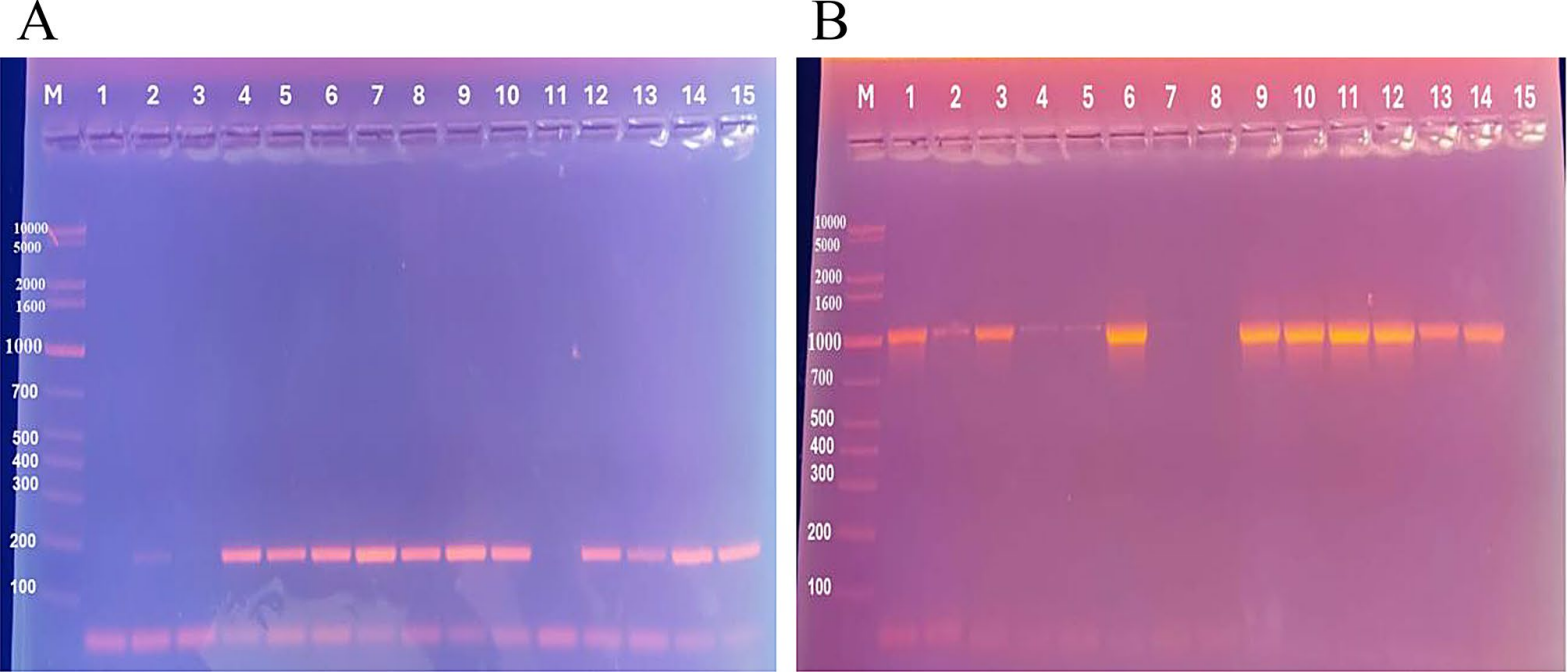
Agarose Gel Electrophoresis of PCR products for *qacE∆1* and *cepA* Genes. Agarose Gel Electrophoresis Showing PCR Bands for *qacE∆1* and *cepA* Genes. Lane M: DNA Molecular Size Marker (100 − 10,000 bp). (**A**): Agarose Gel Electrophoresis for *qacE∆1* gene. Lanes 2, 4–10, and 12–15: Positive for *qacE∆1* with a band size of 190 bp. Lanes 1, 3, and 11: Negative for *qacE∆1*. (**B**): Agarose Gel Electrophoresis for *cepA* gene. Lanes 1–6 and 9–14: Positive for *cepA* with a band size of 1050 bp. Lanes 7, 8, and 15: Negative for *cepA*

The *qacE∆1* gene was detected in 62% of isolates, compared to 72% for *cepA*. Co-expression of both genes occurred in 46% of isolates, while 12% Klebsiella strains lacked both genes (Fig. 3**).** Focusing on the relation between various minimal inhibitory concentrations of Benzalkonium chloride and Chlorhexidine with the presence of *qacE∆1* and *cepA* genes, no significant correlations were seen as demonstrated in Table 3.

Data in Table 4 show in-details the antimicrobial resistance patterns of studied 50 *Klebsiella* strains in relation to the presence of the *cepA* and *qacE∆1* genes revealing that Cefotaxime and Cefoperazone exhibited significant differences (*p* < 0.05) in resistance between *qacEΔ1*-positive and *qacEΔ1*-negative isolates. However, *cepA* presence did not show statistically significant resistance differences across tested antibiotics.

Among *qacE∆1*-positive isolates, 45.1% were multidrug resistant (MDR), 41.9% were extensively drug-resistant (XDR), and 13% were pan-drug resistant (PDR). In comparison, *qacE∆1*-negative isolates demonstrated 52.6% MDR, 36.9% XDR, and 10.5% PDR, with no significant difference in resistance profiles (*p* = 0.874). For *cepA*-positive isolates, 36.1% were MDR, 50% were XDR, and 13.9% were PDR. Conversely, *cepA*-negative isolates exhibited a higher MDR rate (78.6%) but lower rates of XDR (14.3%) and PDR (7.1%) with observed statistical significance (*p* = 0.0249).

Among isolates co-expressing both *qacE∆1* and *cepA* genes, 39.1% were MDR, 47.8% were XDR, and 13.0% were PDR. In comparison, isolates lacking both genes exhibited 33.3% for MDR, 50.0% for XDR, and 16.7% for PDR. Chi-square analysis showed no statistically significant association between gene co-expression or absence and resistance phenotypes, with *p* values of 0.486 and 0.860, respectively. Although *qacE∆1* and *cepA* individually influenced specific resistance patterns, their co-expression or absence did not have a significant impact on the overall resistance profiles of *Klebsiella pneumoniae* isolates as illustrated in Table 5.

**Table 2:** Correlation of minimal inhibitory concentrations (MIC) values of Benzalkonium chloride and chlorhexidine with resistance profiles (MDR, XDR, PDR) in *Klebsiella pneumoniae* isolates (*N* = 50)

Resistance patterns	Benzalkonium chloride MIC (µg/mL)			chlorhexidine MIC (µg/mL)			*p*-values
	Mean	Median	Range	Mean	Median	Range	
**MDR(Multidrug resistance)**	23.00	16.00	8–64	59.67	64.00	8-128	*P-value = 0.005
**XDR(Extensively drug resistance)**	43.60	16.00	8-256	63.00	64.00	4-128	**P-value = 0.670
**PDR(Pan-drug resistance)**	85.33	64.00	32–256	48.00	40.00	8-128	**P1 = 0.459** **P2 = 0.001** **P3 = 0.006**

*p-value: Represents the statistical analysis of benzalkonium chloride MIC values in relation to resistance patterns

**P-value: Represents the statistical analysis of chlorhexidine MIC values in relation to resistance patterns

P1: Compares benzalkonium chloride MIC values between MDR and XDR strains

P2: Compares benzalkonium chloride MIC values between MDR and PDR strains

P3: Compares benzalkonium chloride MIC values between XDR and PDR strains

**Table 3:** Association between the minimal inhibitory concentrations (MIC) of Benzalkonium chloride and chlorhexidine with the presence of biocide resistance genes *QacE∆1* and *CepA* in *Klebsiella pneumoniae* isolates

Biocide resistance genes	MIC of Benzalkonium chloride (µg/mL)														
	Total isolates (*n* = 50)	8		16		32		64		128		256		X2	p value
		NO	%	NO	%	NO	%	NO	%	NO	%	NO	%		
*qacE∆1*															
positive	31	2	6.5	15	48.4	6	19.4	4	12.9	2	6.5	2	6.5	8.48	0.132
negative	19	0	0	9	47.4	10	52.6	-	-	-	-	-	-		
*cepA*															
positive	36	1	2.8	20	55.6	10	27.8	2	5.6	1	2.8	2	5.6	5.57	0.322
negative	14	1	7.1	4	28.6	6	42.9	2	14.3	1	7.1	-	-		
Biocide resistance genes	MIC of Chlorohexidine (µg/mL)														
	Total isolates (*n* = 50)	4		8		16		32		64		128		X2	p value
		NO	%	NO	%	NO	%	NO	%	NO	%	NO	%		
*qacE∆1*															
positive	31	-	-	6	19.4	4	12.9	6	19.4	10	32.3	5	16.1	9.63	0.06
negative	19	1	5.3	-	-	1	5.3	1	5.3	11	57.9	5	26.3		
*cepA*															
positive	36	-	-	3	8.3	4	11.1	6	16.7	16	44.4	7	19.4	0.177	0.68
negative	14	1	7.1	3	21.4	1	7.1	1	7.1	5	35.7	3	21.4		

**Table 4:** Correlation of antibiotic resistance patterns in *Klebsiella pneumoniae* isolates with *QacE∆1* and *CepA* genes

Antimicrobial agent	qacE∆1 positive isolates (NO = 31)		qacE∆1 negative isolates (NO = 19)		cepA positive isolates (NO = 36)		cepA negative isolates (NO = 14)		*P*	*P
	Resistant isolates NO (%)									
	NO	%	NO	%	NO	%	NO	%		
Ampicillin	25	81	17	90	30	83	12	86	1.00	0.38
piperacillin -tazobactam	22	71	12	63	24	67	10	71	0.78	0.76
Amoxicillin -clavulanic	21	68	17	90	28	78	10	71	0.28	0.57
Ampicillin -sulbactam	26	84	15	79	28	78	13	93	0.29	0.60
ceftazidime -avibactam	22	71	13	68	25	69	10	71	0.84	0.90
ceftolozane-tazobactam	20	65	14	74	23	64	11	79	0.90	0.39
Ceftazidime	28	90	17	90	33	92	12	86	1.00	0.43
Cefotaxime	30	97	14	74	32	89	12	86	0.03	0.78
Cefixime	27	87	19	100	32	89	14	100	0.28	0.57
Cefoxitin	23	74	18	95	29	81	12	86	0.13	0.72
cefoperazone	29	94	16	84	32	89	13	93	0.03	1.00
Aztreonam	25	81	16	84	31	86	10	71	0.41	0.18
Meropenem	7	23	1	5	5	14	3	21	0.13	0.83
Imipenem	7	23	8	42	12	33	3	21	0.13	0.32
Ertapenem	15	48	4	21	14	39	5	36	0.07	1.00
Amikacin	25	81	13	68	30	83	8	57	0.38	0.08
Gentamycin	24	77	10	53	27	75	7	50	0.08	0.15
Tetracycline	17	55	11	58	19	53	9	64	0.53	0.82
Doxycycline	18	58	10	53	20	56	8	57	0.77	1.00
Azithromycin	27	87	18	95	33	92	12	86	0.12	0.69
Ciprofloxacin	22	71	17	90	28	78	11	79	0.23	1.00
Levofloxacin	22	71	16	84	26	72	12	86	0.12	0.62
Trimethoprim/ sulfa -methoxazole	16	52	14	74	22	61	8	57	0.28	0.82
For urine specimens (*n* = 19)	*qacE∆1* Positive isolates (NO = 11)		*qacE∆1* negative isolates (NO = 8)		*cepA* positive isolates (NO = 15)		*cepA* negative isolates (NO = 4)		P	*P
	NO	%	NO	%	NO	%	NO	%		
Nitrofurantoin	4	36	5	63	7	47	2	50	0.49	0.87

**Table 5:** Distribution of *QacE∆1* and *CepA* genes among *Klebsiella pneumoniae* isolates and their association with different resistance phenotypes

Klebsiella pneumoniae isolates	MDR		XDR		PDR		X2	*p*-value
	NO	%	NO	%	NO	%		
*qacE∆1* positive isolates (NO = 31)	14	45.1	13	41.9	4	13	0.2688	0.874
*qacE∆1* negative isolates (NO = 19)	10	52.6	7	36.9	2	10.5		
*Cep A* positive isolates (NO = 36)	13	36.1	18	50	5	13.9	7.3826	0.0249
*CepA* negative isolates (NO = 14)	11	78.6	2	14.3	1	7.1		
Co-existence of *qacE∆1* and *cepA)*NO = 23)	9	39.1	11	47.8	3	13	1.389	0.486
Absence of *qacE∆1* and *cepA*(NO = 6)	2	33.3	3	50	1	16.7	0.6	0.86

## Discussion

*Klebsiella pneumoniae* is a common opportunistic pathogen resistant to most antibiotics. The rise of disinfectant resistance further threatens health by reducing biocide effectiveness. Disinfection practices and antibiotic use vary by region, leading to different resistance mechanisms. Local epidemiological studies are decisive for effective infection control, yet such studies are limited in our area [7].

In this study, *Klebsiella pneumoniae* accounted for 19.6% of detected pathogens, emerging as the most frequently isolated microbe. Notably, higher isolation rates (24.5%) were reported by Mohamed et al. [15]. Also the study of El-Sherbiny, 2024 [16] detected *Klebsiella pneumonia* by 30.85%. Globally, Antonelli et al. [17] identified Gram-negative bacilli as the predominant group in Italy, with *Klebsiella pneumoniae* comprising 10.4% of isolates. Similarly, Mobarak-Qamsari and colleagues reported *Klebsiella pneumoniae* as the leading Gram-negative pathogen among ICU patients in Tehran, accounting for 41.5% of cases [18].

The findings of the current study revealed high resistance rates of *Klebsiella pneumoniae* to older antibiotics, including ampicillin, cephalosporins, macrolides, quinolones, aminoglycosides, and monobactams, with resistance exceeding 80%. While piperacillin-tazobactam and ceftazidime-avibactam showed about 70% resistance. However, carbapenems remained effective, with susceptibility rates exceeding 70%. These results were consistent with multiple global and regional studies [7, 16, 19, 20]. Conversely, Itani et al. [21] and Worku et al. [22] reported significantly lower resistance rates for these antibiotics, suggesting a lower resistance justified by variability of resistance patterns across regions reflecting the importance of local surveillance data.

This work revealed relatively low resistance to colistin among *Klebsiella pneumoniae* isolates, with only 6 isolates (12%) classified as resistant, while the majority (44 isolates, 88%) were intermediate-sensitive. These findings were aligned with the low resistance rates reported by Itani et al. [21] in Lebanon (7.7%) and Ibrahim [23] in Saudi Arabia (16.3%). Similarly, Rabie & Abdallah [24] from Egypt reported a resistance rate of 17.2%. In contrast, higher resistance rates were observed in other studies, including Jalal et al. [25] with 21.5% and Abozahra et al. [26] with 39%, highlighting regional and environmental variations in resistance trends.

In the current study, nearly half (48%) of the isolates were classified as MDR, while 40% exhibited XDR, and 12% showed PDR. Similarly, Ahmed et al. [27] in Egypt and Ogefere & Idoko [28] in Nigeria reported MDR prevalence of about 50%. In Iran, Esfahanian et al. [29] observed a lower MDR prevalence of 35% and an XDR rate of 22%, though PDR was not reported. Meanwhile, Itani et al. [21] reported that 61% of *Klebsiella pneumoniae* isolates were MDR, 7.3% were XDR, while 0.5% were PDR.

Our findings revealed that *Klebsiella* isolates exhibited multiple antibiotic resistance (MAR) index values ranging from 0.24 to 1.0, reflecting a broad spectrum of resistance levels as described by Ahmed et al. [27]. These patterns were aligned with several global observations. Ayandele et al. [30] reported MAR indices ranging from 0.29 to 1.0 among *Klebsiella pneumoniae* isolates from clinical settings in Nigeria, while Ghenea et al. [31] found MAR indices frequently exceeding 0.8 in isolates from intensive care and surgical units, reflecting the selective pressure of intensive antibiotic use. Ogefere & Idoko (2024) documented MAR indices from 0.42 to 1.00 in *Klebsiella pneumoniae* isolates from a tertiary hospital with higher resistance levels among inpatients compared to outpatients, emphasizing the resistance risks associated with clinical settings.

This current study revealed a significant direct correlation between antibiotic resistance levels and Benzalkonium chloride MIC values. In contrast, a p-value of 0.67 indicated no significant difference in Chlorhexidine MICs across different resistance profiles. In Iraq, Hassan et al. [32] attributed this variability to the presence of various virulence factors, such as biofilm formation and the acquisition of biocide resistance genes. Zhao et al. [33] reported statistically significant differences in Benzalkonium MIC values between extensively drug-resistant (XDR) and non-XDR *Klebsiella pneumoniae* strains, reinforcing the connection between antibiotic resistance and reduced susceptibility to disinfectants. These findings highlighted the need for tailored biocide application strategies to mitigate resistance development.

In this work, *qacEΔ1* was detected in 62% of the isolates, while *cepA* was seen in 72% of them. Both genes were co-expressed in 46% of isolates. A similar study by Liu et al. [7] in China found *qacEΔ1* in 64.9% and *cepA* in 93.2% of *Klebsiella pneumoniae* isolates, with 62.2% of isolates co-expressing both genes. Mikhaylovskaya et al. [34] reported *qacEΔ1* and *cepA* prevalence in MDR *Klebsiella pneumoniae* isolates at 54% and 72%, respectively. In contrast, Chen et al. [35] found *qacEΔ1* in 41.7% of isolates, with *cepA* detected in over 80%, highlighting regional variations in the prevalence of these resistance genes, which may be influenced by differing biocide usage protocols.

The distribution of *qacEΔ1* showed no statistically significant association with resistance patterns (p-value = 0.874). However, *cepA* was significantly linked to an increased occurrence of XDR pattern (p-value = 0.0249), suggesting its role in contributing to extensive drug resistance. Afshar-Yavari et al. [36] did not find a significant relationship between *cepA* and resistance patterns in *Klebsiella pneumoniae*. Furthermore, there is a lack of sufficient studies exploring the relationship between *qacEΔ1* and resistance patterns in *Klebsiella pneumoniae* isolates.

This study also examined the association between the presence of *qacEΔ1* and *cepA* genes and antibiotic resistance. Our findings showed significant differences in resistance to Cefotaxime and Cefoperazone in *qacEΔ1*-positive isolates (*p* < 0.05), suggesting a contribution of this gene to increased resistance to mentioned antibiotics. In contrast, no statistically significant differences were observed between *cepA*-positive and *cepA*-negative isolates across tested antibiotics. These results align partially with Mihailovskaya et al. [33], who found no significant correlation between *cepA* and resistance to specific antibiotic classes but noted a strong association between *qacEΔ1* and resistance to cephalosporins, carbapenems, and fluoroquinolones. Liu et al. [7] similarly found that *qacEΔ1* and *cepA-*positive isolates exhibited significant resistance to a broad range of antibiotics.

We also could not find significant association between the biocide resistance genes *qacEΔ1* or *cepA* and the MIC levels of benzalkonium chloride (BAC) and chlorhexidine (CHX). These findings are consistent with those of Vijayakumar et al. [13] in Saudi Arabia, who reported no significant correlation with multidrug-resistant strains of *Klebsiella pneumoniae*. However, contrasting evidence exists in other studies. Mihailovskaya et al. [34] observed that efflux pump genes, including *qacEΔ1* and *cepA*, were associated with elevated MICs for BAC and CHX and these genes were frequently present in clinical isolates of MDR *K. pneumoniae*. Similarly, Afshar-Yavari et al. [36] found a significant relationship between the presence of *cepA* and high MICs for CHX in *K. pneumoniae*. These differing results highlight the complexity of biocide resistance mechanisms and the potential influence of environmental and clinical factors on the activity of efflux pumps.

Rational administration of biocides has been shown to increase the minimum inhibitory concentration of the tested agents. Applying disinfectants at concentrations lower than the inhibitory concentration may contribute to the spread of bacteria resistant to disinfectants, thereby increasing the risk of infection transmission to patients and the environment [37].

## Conclusions

This study highlighted the extensive antibiotic resistance in *Klebsiella pneumoniae*, characterized by significant resistance to older antibiotics with a notable prevalence of multidrug resistant (MDR) and extensively drug resistant (XDR) strains. While the *qacEΔ1* and *cepA* genes were frequently detected, no strong correlation was established between presence of these genes and biocide resistance. The variability in resistance patterns underscored the importance of understanding regional epidemiological data. These findings emphasized the critical need for effective infection control measures, including the rational use of biocides at concentrations ≥ 64 µg/mL and the mandatory identification of resistant strains to mitigate the risk of bacterial spread.

### Recommendations

Further research is needed to fully understand the role of *qacEΔ1* and *cepA* in resistance to BAC and CHX. Hospitals should enhance surveillance, investigate efflux pump genes, optimize biocide use, enforce strict antibiotic stewardship, and improve disinfection protocols to strengthen the prevention and treatment of multidrug-resistant *Klebsiella pneumoniae*. Additionally, tailored biocide strategies and focused research on bacterial resistance mechanisms to antibiotics and biocides are essential for developing effective prevention and treatment rules.

## Electronic supplementary material

Below is the link to the electronic supplementary material.

Supplementary Material 1

## Data Availability

Available on request from authors.

## Abbreviations

CLSI: Clinical and Laboratory Standards Institute
MIC: Minimum inhibitory concentration
MDR: multidrug resistant
XDR: extensively drug resistant
PDR: pan-drug resistant
MAR: multiple antimicrobial resistance index
