# Supplementary material for: Chlorhexidine and benzalkonium chloride: promising adjuncts in combating multidrug resistant Klebsiella pneumoniae in healthcare settings
Source: BMC Infect Dis. 2025 May 7;25:670. doi: 10.1186/s12879-025-10980-w (PMC12057036; doi:10.1186/s12879-025-10980-w)
Supplement: Supplementary file 1 — Supplementary Material 1 [file 12879_2025_10980_MOESM1_ESM.docx]

**Supplementary tables**

**Table (1- S ):** **Distribution of Isolated Microorganisms In Positive Growth Cultures (NO=261)**

| Isolated microorganisms | **NO** | **%** |
| --- | --- | --- |
| **Single microorganism (229)** | | |
| Gram-positive cocci | 66 | 25% |
| *Staph. aureus* | 27 | 10.34% |
| *Coagulase-negative staph. (CoNS)* | 19 | 7.28% |
| *Streptococcus pneumoniae* | 7 | 2.68% |
| *Enterococcus* spp*.* | 13 | 4.98% |
| Gram-negative bacilli | 149 | 57% |
| *E.coli* | 37 | 14.18% |
| *Klebsiella* spp. | 43 | 16.48% |
| *Enterobacter* spp. | 6 | 2.30% |
| *Proteus* spp. | 8 | 3.07% |
| *Acinetobacter* spp. | 29 | 11.11% |
| *Pseudomonas* spp. | 26 | 9.96% |
| Fungi | 14 | 5.36% |
| *Candida* spp*.* | 14 | 5.36% |
| **Mixed microorganisms (32)** | | |
| *Klebsiella* spp. *+ Pseudomonas* spp. | 4 | 1.53% |
| *Klebsiella spp. +Staph.aureus* | 2 | 0.77% |
| *Klebsiella spp.+Acinetobacter* spp. | 1 | 0.38% |
| *E.coli+ Pseudomonas* spp. | 2 | 0.77% |
| *E.coli+ Staph. Aureus* | 4 | 1.53% |
| *E.coli +Enterococcus spp.* | 2 | 0.77% |
| *Enterobacter spp.+ Pseudomonas* spp. | 3 | 1.15% |
| *Enterobacter + Acinetobacter* spp | 3 | 1.15% |
| *Enterobacter + Coagulase-negative staph.* | 2 | 0.77% |
| *Pseudomonas* spp*. + Staph. aureus* | 3 | 1.15% |
| *Pseudomonas* spp. *+ Coagulase-negative staph.* | 2 | 0.77% |
| *Acinetobacter* spp. *+ Staph. aureus* | 3 | 1.15% |
| *Acinetobacter* spp. *+Coagulase-negative staph.* | 1 | 0.38% |
| Total positive cultures | 261 | 100% |

**Table (2-S): Antimicro****bial susceptibility pattern of *Klebsiella pneumoniae* isolates (NO= 50) by disk diffusion method:**

| **Antimicrobial group** | **Antimicrobial agent** | **Abbreviation** | **Disk Content** | **S** | | **I** | | **R** | |
| --- | --- | --- | --- | --- | --- | --- | --- | --- | --- |
|  |  |  |  | **NO** | **%** | **NO** | **%** | **NO** | **%** |
| **Penicillin** | Ampicillin | AMP | 10μg | 4 | 8 | 4 | 8 | 42 | 84 |
| **β--lactam/β-lactamase inhibitor combination** | Piperacillin - tazobactam | PIT | 100/10μg | 13 | 26 | 3 | 6 | 34 | 68 |
|  | Amoxicillin -clavulanic | AMC | 20 /10μg | 7 | 14 | 5 | 10 | 38 | 76 |
|  | Ampicillin -sulbactam | A/S | 10/10μg | 5 | 10 | 4 | 8 | 41 | 82 |
|  | ceftazidime -avibactam | CZA | 30/20μg | 15 | 30 | - | - | 35 | 70 |
|  | ceftolozane-tazobactam | C/T | 30/10μg | 10 | 20 | 6 | 12 | 34 | 68 |
| **Cephalosporins** | ceftazidime | CAZ | 30μg | 5 | 10 | - | - | 45 | 90 |
|  | Cefotaxime | CTX | 30μg | 4 | 8 | 2 | 4 | 44 | 88 |
|  | Cefixime | CFM | 5μg | - | - | 4 | 8 | 46 | 92 |
|  | Cefoxitin | CX | 30μg | 9 | 18 | - | - | 41 | 82 |
|  | Cefoperazone | CPZ | 75μg | 2 | 4 | 3 | 6 | 45 | 90 |
| **Monobactams** | Aztreonam | AT | 30μg | 8 | 16 | 1 | 2 | 41 | 82 |
| **Carbapenems** | Meropenem | MRP | 10μg | 40 | 80 | 2 | 4 | 8 | 16 |
|  | Imipenem | IPM | 10μg | 35 | 70 | - | - | 15 | 30 |
|  | Ertapenem | ETP | 10μg | 31 | 62 | - | - | 19 | 38 |
| **Aminoglycosides** | Amikacin | AK | 30μg | 11 | 22 | 1 | 2 | 38 | 76 |
|  | Gentamycin | GEN | 10μg | 15 | 30 | 1 | 2 | 34 | 68 |
| **Tetracyclines** | Tetracycline | TE | 30μg | 21 | 42 | 1 | 2 | 28 | 56 |
|  | Doxycycline | DO | 30μg | 22 | 44 | - | - | 28 | 56 |
| **Macrolides** | Azithromycin | AZM | 15μg | 5 | 10 | - | - | 45 | 90 |
| **Quinolones** | Ciprofloxacin | CIP | 5μg | 8 | 16 | 3 | 6 | 39 | 78 |
|  | Levofloxacin | LE | 5μg | 11 | 22 | 1 | 2 | 38 | 76 |
| **Folate metabolism antagonist** | Trimethoprim/ sulfa -methoxazole | COT | 1.25/23.75μg | 19 | 38 | 1 | 2 | 30 | 60 |
| **For urine specimens (NO=19(** | | | | | | | | | |
| **Nitrofurans** | Nitrofurantoin | NIT | 300 μg | 6 | 31.6 | 4 | 21.1 | 9 | 47.3 |

**Table (3-S): Frequency of multi-drug, extensive-drug and pan-drug resistance among klebsiella** **pneumoniae isolates (NO =50):**

| ***Klebsiella* isolates** | **Multidrug resistance (MDR)**  **NO (%)** | **Extensively drug resistance (XDR)**  **NO (%)** | **Pan-drug resistance (PDR)**  **NO (%)** |
| --- | --- | --- | --- |
| **Total (NO =50)** | **24 (48%)** | **20 (40%)** | **6 (12%)** |

- **Multidrug resistance (MDR): Resistant to ≥ 1 agent in ≥ 3 antimicrobial categories.**
- **Extensively drug resistant (XDR): Resistant to ≥ 1 agent in all but susceptible for ≤ 2 categories.**
- **Pan-drug resistant (PDR): Resistant to all antimicrobial agents listed**

**Table (4-S): Antimicrobial Resistance Patterns and Multiple Antimicrobial Resistance (MAR) Index for *Klebsiella pneumoniae* Isolates:**

| **CODE** | **Resistance Pattern** | ***MAR_**  **Index** | **No of isolates** | **Percentage**  **%** | **Character of resistant strain** |
| --- | --- | --- | --- | --- | --- |
| ***K1** | AMP, CTX, CPZ, AT , AZM , COT | 0.24 | 1 | 2 | MDR |
| ***K2** | A/S, CAZ, CTX, CPZ, AT , AK, AZM , NIT | 0.32 | 1 | 2 | MDR |
| ***K3** | AMP, AMC, CAZ, CTX, CFM, CX, CPZ, AT , AZM | 0.36 | 1 | 2 | MDR |
| ***K4** | AMP, A/S, CFM, AT , AK, GEN, CIP, LE, AZM | 0.36 | 1 | 2 | MDR |
| **K5** | AMP, CAZ, CTX, CFM, CX, CPZ, AK, GEN, AZM | 0.36 | 1 | 2 | MDR |
| ***K6** | AMP, A/S, CAZ, CX, CZA, AT , CIP, LE, AZM | 0.38 | 1 | 2 | MDR |
| ***K7** | AMP, A/S, CAZ, CX, AT , TE , CIP, LE, COT | 0.38 | 1 | 2 | MDR |
| ***K8** | AMC, CTX, CFM, CZA, IPM, AK, GEN, CIP, AZM , COT | 0.40 | 1 | 2 | MDR |
| ***K9** | AMP, AMC, CTX, CFM, CPZ, AK, GEN, LE, AZM , NIT | 0.40 | 1 | 2 | MDR |
| **K10** | AMP, A/S, CAZ, CTX, CFM, CPZ, CZA, AZM , COT, NIT | 0.40 | 1 | 2 | MDR |
| ***K11** | AMP, AMC, CFM, CX, CPZ, C/T, CIP, LE, AZM , NIT | 0.40 | 1 | 2 | MDR |
| ***K12** | PIT , CAZ, CTX, CFM, CX, CPZ, AK, GEN, CIP, LE, COT | 0.44 | 1 | 2 | MDR |
| ***K13** | AMP, CAZ, CTX, , CFM, CX, CPZ, CZA, AT , AK, AZM , NIT | 0.44 | 1 | 2 | MDR |
| ***K14** | AMP, PIT , A/S, CAZ, CTX, CFM, CX, CPZ, CZA, AT , AZM | 0.44 | 1 | 2 | MDR |
| ***K15** | PIT , AMC, CAZ, CTX, CFM, CZA, C/T, AK, GEN, CIP, LE, AZM | 0.48 | 1 | 2 | MDR |
| **K16** | A/S, CAZ, CFM, CX, CPZ, CZA, C/T, TE , DO, CIP, LE, AZM | 0.50 | 1 | 2 | MDR |
| **K17** | AMP, PIT , AMC, A/S, CAZ, CTX, CFM, CX, CPZ, AT , AK, GEN, COT | 0.52 | 1 | 2 | MDR |
| ***K18** | AMP, A/S, CAZ, CTX, CFM, CPZ, CZA, C/T, AT , TE , CIP, LE, AZM | 0.54 | 1 | 2 | MDR |
| ***K19** | AMP, AMC, A/S, CAZ, CTX, CFM, CX, CPZ, CZA, C/T, AK, TE , DO, AZM | 0.58 | 1 | 2 | MDR |
| **K20** | AMC, A/S, CAZ, CTX, CFM, CX, CPZ, CZA, AT , IPM, AK, GEN, CIP, AZM , NIT | 0.60 | 1 | 2 | MDR |
| **K21** | AMP, PIT , AMC, A/S, CAZ, CTX, CFM, CX, CPZ, C/T, AT , CIP, LE, AZM , COT | 0.63 | 1 | 2 | MDR |
| **K22** | PIT , AMC, A/S, CAZ, CTX, , CFM, CX, CPZ, C/T, AT , AK, GEN, CIP, LE, AZM , COT | 0.67 | 1 | 2 | MDR |
| **K23** | AMP, PIT , AMC, A/S, CAZ, CTX, CFM, CX, CPZ, CZA, AT , ETP, AK, DO, CIP, AZM | 0.67 | 1 | 2 | MDR |
| **K24** | PIT , AMC, A/S, CAZ, CTX, CFM, CPZ, C/T, AT , ETP, AK, GEN, TE , DO, CIP, LE, AZM | 0.71 | 1 | 2 | MDR |
| **K25** | AMP, PIT , AMC, A/S, CAZ, CTX, CFM, CX, CPZ, CZA, C/T, AT , TE , DO, CIP, LE, AZM , COT | 0.75 | 2 | 4 | XDR |
| **K26** | AMP, PIT , AMC, A/S, CAZ, CTX, CFM, CX, CPZ, CZA, C/T, AT , AK, GEN, TE , DO, CIP, LE, AZM | 0.79 | 3 | 6 | XDR |
| **K27** | AMP, PIT , AMC, A/S, CAZ, CTX, CFM, CX, CPZ, CZA, C/T, AT , AK, GEN, TE , DO, CIP, LE, COT | 0.79 | 1 | 2 | XDR |
| **K28** | AMP, PIT , AMC, A/S, CAZ, CTX, CFM, CX, CPZ, CZA, C/T, AT , AK, GEN, TE , DO, LE, AZM , COT | 0.79 | 1 | 2 | XDR |
| **K29** | AMP, PIT , AMC, A/S, CAZ, CTX, CFM, CX, CPZ, CZA, C/T, AT , AK, GEN, TE , CIP, LE, AZM , COT | 0.79 | 1 | 2 | XDR |
| **K30** | AMP, PIT , AMC, A/S, CAZ, CTX, CFM, CX, CPZ, CZA, C/T, AT , ETP, AK, GEN, CIP, LE, AZM , COT | 0.79 | 1 | 2 | XDR |
| **K31** | AMP, PIT , AMC, A/S, CAZ, CTX, CFM, CX, CPZ, CZA, C/T, AT , AK, GEN, TE , DO, CIP, LE, AZM , COT | 0.83 | 1 | 2 | XDR |
| **K32** | AMP, PIT , AMC, A/S, CAZ, CTX, CFM, CX, CPZ, CZA, C/T, AT , ETP, AK, GEN, TE , DO, CIP, LE, AZM | 0.83 | 1 | 2 | XDR |
| **K33** | AMP, PIT , AMC, A/S, CAZ, CFM, CX, CPZ, CZA, C/T, AT , IPM, ETP, AK, GEN, TE , DO, CIP, LE, AZM , COT | 0.88 | 1 | 2 | XDR |
| **K34** | AMP, PIT , AMC, A/S, CAZ, CTX, CFM, CX, CPZ, CZA, C/T, AT , ETP, AK, GEN, TE , DO, CIP, LE, AZM , COT | 0.88 | 2 | 4 | XDR |
| **K35** | AMP, PIT , AMC, A/S, CAZ, CTX, CFM, CX, CPZ, CZA, C/T, AT , IPM, ETP, AK, GEN, DO, CIP, LE, AZM , COT | 0.88 | 2 | 4 | XDR |
| ***K36** | AMP, PIT , AMC, A/S, CAZ, CTX, CFM, CX, CPZ, CZA, C/T, AT , IPM, ETP, AK, GEN, TE , DO, CIP, LE, AZM , COT, NIT | 0.92 | 2 | 4 | XDR |
| ***K37** | AMP, PIT , AMC, A/S, CAZ, CTX, CFM, CX, CPZ, CZA, C/T, AT , MRP, IPM, ETP, AK, GEN, TE , DO, CIP, LE, COT, NIT | 0.92 | 1 | 2 | XDR |
| ***K38** | AMP, PIT , AMC, A/S, CAZ, CTX, CFM, CX, CPZ, CZA, C/T, AT , MRP, IPM, ETP, AK, GEN, TE , DO, CIP, LE, AZM , COT | 0.92 | 1 | 2 | XDR |
| **K39** | AMP, PIT , AMC, A/S, CAZ, CTX, CFM, CX, CPZ, CZA, C/T, AT , MRP, IPM, ETP, AK, GEN, TE , DO, CIP, LE, AZM , COT, colistin | 1.00 | 6 | 12 | PDR |

- MDR: multiple drug resistant, XDR: extensively drug resistant, PDR: pan-drug resistant
- MAR (multiple antimicrobial resistance index): ratio of number of antibiotics to which organism is resistant / total number of antibiotics to which organism is exposed.
- Isolate number: number of isolates have resistant to same antibiotics.
- Percentage: is (the percent of isolate number / number of total isolates) x 100
- MDR: resistant to ≥ 1 agent in ≥ 3 antimicrobial categories of tested antibiotics
- XDR: resistant to ≥ 1 agent in all but susceptible for ≤ 2 categories of tested antibiotics
- PDR: resistant to all antimicrobial agents of tested antibiotics.
- The antimicrobial agents and their abbreviations are as follows: Ampicillin (AMP), Piperacillin-Tazobactam (PIT), Amoxicillin-Clavulanic (AMC), Ampicillin-Sulbactam (A/S), Ceftazidime-Avibactam (CZA), Ceftolozane-Tazobactam (C/T), Ceftazidime (CAZ), Cefotaxime (CTX), Cefixime (CFM), Cefoxitin (CX), Cefoperazone (CPZ), Aztreonam (AT), Meropenem (MRP), Imipenem (IPM), Ertapenem (ETP), Amikacin (AK), Gentamycin (GEN), Tetracycline (TE), Doxycycline (DO), Azithromycin (AZM), Ciprofloxacin (CIP), Levofloxacin (LE), Trimethoprim/Sulfamethoxazole (COT) and Nitrofurantoin(NIT).

*K= represents urine isolates

- **Note:All antibiotics are tested using disc diffusion method except colistin is tested using agar dilution method**
